# Highly improved pH-Responsive anticancer drug delivery and T2-Weighted MRI imaging by magnetic MOF CuBTC-based nano/microcomposite

**DOI:** 10.3389/fmolb.2023.1071376

**Published:** 2023-04-04

**Authors:** Zahra Gharehdaghi, Seyed Morteza Naghib, Rahmatollah Rahimi, Atin Bakhshi, Amirhosein Kefayat, Armin shamaeizadeh, Fatemeh Molaabasi

**Affiliations:** ^1^ Department of Chemistry, Iran University of Science and Technology, Tehran, Iran; ^2^ Nanotechnology Department, School of Advanced Technologies, Iran University of Science and Technology (IUST), Tehran, Iran; ^3^ Biomaterials and Tissue Engineering Research Group, Department of Interdisciplinary Technologies, Breast Cancer Research Center, Motamed Cancer Institute, ACECR, Tehran, Iran

**Keywords:** magnetic, metal-organic framework, controlled release, pH-responsive doxorubicin release, magnetic resonance imaging, multifunctional nanocomposite

## Abstract

Cu-BTC framework has received a considerable attention in recent years as a drug carrier candidate for cancer treatment due to its unique structural properties and promising biocompatibility. However, its intrinsic deficiency for medical imaging potentially limits its bioapplications; To address this subject, a magnetic nano/microscale MOF has been successfully fabricated by introducing Fe_3_O_4_ nanoparticles as an imaging agent into the porous isoreticular MOF [Cu_3_(BTC)_2_] as a drug carrier. The synthesized magnetic MOFs exhibits a high loading capacity (40.5%) toward the model anticancer DOX with an excellent pH-responsive drug release. The proposed nanocomposite not only possesses large surface area, high magnetic response, large mesopore volume, high transverse relaxivity (*r* ^2^) and good stability but also exhibits superior biocompatibility, specific tumor cellular uptake, and significant cancer cell viability inhibitory effect without any targeting agent. It is expected that the synthesized magnetic nano/microcomposite may be used for clinical purposes and can also serve as a platform for photoactive antibacterial therapy ae well as pH/GSH/photo-triple-responsive nanocarrier.

## Introduction

An important challenge for cancer therapy is to design a smart nanocarrier with pH-sensitive drug-releasing property and high loading capacity of therapeutic molecules like chemotherapy agents while being able to act as a contrast agent for making a multimodal drug delivery system (DDS) (Chowdhuri et al., 2016a). Porous magnetic microspheres with a porous shell and a magnetic core have been of great interest due to the shell and core functions which induce desired properties, including high drug loading efficacy, considerable biocompatibility, and controllable drug release. In particular anticancer DDS, magnetic nanomaterials possess other diverse properties relation to the photothermal activity, magnetically targeted hyperthermia, and MRI contrast agent resulting in the capability to real-time monitoring tumor tissue treatment (Sun et al., 2008; Liu et al., 2011). In this regard, a variety of porous magnetic core–shell structures include carbon materials, polymers, mesoporous oxides, and zeolites. On the other hand, issues such as long fabrication process, low surface area and high reaction temperature, have resulted in high fabrication cost and low scalability (Deng et al., 2009).

As an efficient porous material, Metal Organic Frameworks (MOFs) has received a highly interest in many areas including catalysis (Lee et al., 2009), gas separation and storage (Rosi et al., 2003), drug delivery (Horcajada et al., 2010), biosensing (Allendorf et al., 2009), and especially developing DDSs (Horcajada et al., 2010) due to their high porosity and surface area, various available structures, adjustable pore sizes and functionalities (Eddaoudi et al., 2001). Compared to the typical porous nanomaterials (e.g., silica), the synthesis of MOFs is simpler and more efficient (Wu et al., 2014). Moreover, MOFs can carry metal nanoparticles in for delivering organic materials and drugs with therapeutic purposes (Dhakshinamoorthy and Garcia, 2012), act as a pH/GSH/photo-triple-responsive carrier in developing multimodal chemo/photothermal/chemodynamic therapy (Lou et al., 2022a), serve as an artificial enzyme to create a pH sensitive (Mandal et al., 2022) (Lou et al., 2022b) (Xu et al., 2022) DDS and as a photoactive dual-cationic covalent MOF to construct a cation/photothermal/NO antibacterial therapy system (Luo et al., 2022a) (Luo et al., 2022b). Ferey and coworkers first studied the ability of MIL-100(Fe) and MIL-101(Cr) to encapsulate ibuprofen molecules for sustained drug delivery (Horcajada et al., 2006). Furthermore, Rahimi et al. used porous multifunctional GO/Cu (II)-porphyrin MOF biocompatible nanocomposite (CuG) to produce a pH-responsive drug carrier to treat breast cancer (Gharehdaghi et al., 2021). Recently, metal organic frameworks with core–shell structures have been also employed in biomedicine and optic fields. Studies on MOF-based core–shell (e.g., MOF@MOF microporous core-shell architectures (Koh et al., 2009), MOF@SiO_2_ nanocomposites (Rieter et al., 2007), MOF-cored molecular imprinted polymers (Qian et al., 2011) and polystyrene@MOF photonic films (Wu et al., 2011) are very limited, and porous magnetic MOF-based core–shell structures have not been significantly explored.

Regarding MOFs with MRI property could be referred to Lin’s group that has synthesized a novel MRI contrast agent based on Gd-MOFs (Rieter et al., 2006). Gd-based MOFs had a high longitudinal relaxivity though the leaching Gd^3+^ ions caused nephrogenic systemic fibrosis (Broome et al., 2007), which challenges their clinical applications. Magnetic nanoparticles may be embedded into MOFs to overcome the issue and maximize drug encapsulation capacity (Vakilinezhad et al., 2018). Iron oxide nanoparticles are widely used in MRI as they can shorten transverse relaxation time and also due to biocompatibility (Lee and Hyeon, 2012). Therefore, Fe_3_O_4_ magnetic nanoparticles may be used to produce multifunctional biocompatible MOF-based composites with high drug load capacity and relaxivity. In this regard, researchers produced multifunctional Fe_3_O_4_@PAA/AuNCs/ZIF-8 NPs (Bian et al., 2015) and RITCFe_3_O_4_@IRMOF-3/FA NPs (Chowdhuri et al., 2016b) by coating Fe_3_O_4_ with different types of Zn-based MOFs for cell imaging and drug delivery.

**SCHEME 1:**
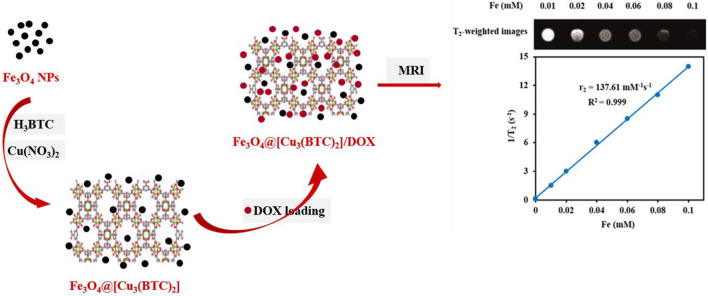
Schematic demonstration of the stepwise synthesis process of Fe_3_O_4_@[Cu_3_(BTC)_2_] and DOX loading into the Fe_3_O_4_@[Cu_3_(BTC)_2_] nanocarrier.

In this study, Fe_3_O_4_at [Cu_3_ (BTC)_2_] magnetic metal organic framework composite was produced using [Cu_3_(BTC)_2_] and Fe_3_O_4_ (Scheme 1). Benzene-1,3,5-tricarboxylate is referred to as BTC and [Cu_3_ (BTC)_2_], is called “HKUST-1”. [Cu_3_(BTC)_2_] has an excellent chemical and solvent stability and therefore has been greatly used for drug delivery. The Cu (II)-cluster coordination and linear ligands form a rigid cubic 3D porous structure with octahedral and tetrahedral cavities. Properties such as numerous open cavities, presence of Cu-O clusters, amphiphilic character and metal sites have made [Cu_3_(BTC)_2_] a candidate for capturing and releasing a variety of anticancer agents. These properties are a result of the coordination bonds of the DOX hydroxyl groups with Cu II) in [Cu_3_(BTC)_2_] (Chui et al., 1999). Moreover, the DOX loaded Fe_3_O_4_@[Cu_3_(BTC)_2_] has shown a sustainable and pH-responsive drug meanwhile having the T_2_-MR contrast property of Fe_3_O_4_ nanospheres. As a result, Fe_3_O4@[Cu_3_(BTC)_2_] composites have high drug loading capacity, low cytotoxicity and high transverse relaxivity, which have made them a candidate for theranostic applications.

## Experimental section

**Materials.** Benzene-1,3,5-tricarboxylic acid (H_3_BTC), ferric chloride hexahydrate (FeCl_3_.6H_2_O), copper II) nitrate trihydrate (Cu (NO_3_)_2_.3H_2_O, 99%), ethylene glycol (EG), sodium acetate (CH_3_COONa), ethanol (C_2_H_6_O, 99%), and phosphate buer (PBS pH 5, 6 and 7.4) was supplied from Merck. Doxorubicin was supplied from Ebewe Pharma Company. Dulbecco’s modified Eagle’s medium (DMEM), trypsin/EDTA and FBS were supplied from Gibco (Grand Island, NY, United States of America). All solutions were prepared using deionized water.

**Synthesis of MAA-Fe**_**3**_ **O**_**4**_ **nanoparticles.** Fe_3_O_4_ particles were produced using solvothermal process. 2.7 g of FeCl_3_.6H_2_O and 4.8 g of NaAc were stirred in 75 ml of ethylene glycol for 30 min using a magnetic stirrer. The mixture was poured in a sealed Teflon-lined stainless-steel container and heated to a temperature of 200°C in an autoclave for 16 h. Then, the Teflon lined container was cooled down to reach room temperature. The resulted Fe_3_O_4_ nanospheres were collected, washed several times using ethanol, and dried under vacuum conditions at a temperature of 50°C (Zeng et al., 2012). 0.5 g Fe_3_O_4_ was added to a 100 ml solution of mercapto-acetic acid in ethanol (2.9 mM) and then was collected by an external magnetic field and finally washed to obtain Mercapto-acetic acid (MAA)-functionalized Fe_3_O_4_ nanoparticles (Wang et al., 2013).

**Synthesis of Fe**_**3**_ **O**_**4**_ **at[Cu**_**3**_ **(BTC)**_**2**_ **] magnetic nanocomposites.** Firstly, 0.25 g of MAA- Fe_3_O_4_ particles were dissolved in 150 ml of ethanol and 1.83 g of Cu (NO_3_)_2_.6H_2_O was added to it and treated using ultrasonic for 30 min. Further, 100 ml of 0.875 g H_3_BTC was added to previous solution with a rate of 1 ml/min under mechanical stirring for 3 h. The Fe_3_O_4_@[Cu_3_(BTC)_2_] particles were extracted from the mixture using a magnetic field, rinsed with ethanol three times, and then dried at a temperature of 60°C and vacuum conditions (Zhao et al., 2015).

**Characterization.** XRD method was used to assess the phase purity of the synthesized products. A Philips PW 1730 X-ray diffractometer (Philips PW 1730/10, Holland) with Cu-Kα radiation was used for this purpose. FTIR test was carried out with IR spectrometer (8500S SHIMADZU, Japan) to identify functional groups. BET method was used to observe the surface area of the materials from N_2_ adsorption and desorption. The measurements were observed on a Micrometritics ASAP2020 system (ASAP 2020; Japan). The distribution of the pore size was found from desorption branches of the N_2_ isotherms with BJH method. Vibrating sample magnetometer (VSM, MDKB, Magnetic Daghigh Kavir Co., Iran) was applied to observe the magnetic properties of as-prepared nanocomposites at room temperature. The transverse relaxivity times and T_2_-weighted MR images were taken with a Siemens Prisma 3.0 TMR scanner (Siemens 3.0 T MAGNETOM Prisma, Germany) having a gradient strength up to 80 mT/m under the following sequence (multi spin-echo, FOV of 100 × 100 mm, TR/TE = 2000/60 m, slices = 1, and a matrix of 192 × 256, 0.55 T, 32.0°C). Tescan Mira3 FE-SEM was used to observe the morphology of synthesized samples (TESCAN MIRA3, Australia). The UV-Visible characterization of the samples was carried out by Shimadzu UV-1700 (Shimadzu UV-1700, Japan) spectrophotometer.

***In vitro* loading and release of DOX.** DOX was loaded into Fe_3_O_4_@[Cu_3_(BTC)_2_] nano/microcomposites *via* adding 2 ml of 2 mg/ml DOX solution to a 5 mg of Fe_3_O_4_@[Cu_3_(BTC)_2_] (2.5 mg/ml). The solution was mixed for 24 h at room temperature using a 180-rpm shaker in a dark room. The Fe_3_O_4_@[Cu_3_(BTC)_2_] particles loaded with drug and then were removed from the solution using magnetic decantation. The DLC of the magnetic nanocomposite was determined from UV-Vis absorbance at 480 nm using to the following formula:

$$DLC\;\left(wt.\;\%\right)=weight\;of\;loaded\;DOX\;/\;\left(total\;weight\;of\;loaded\;DOX\;and\;{Fe}_{3}O_{4}@\left[{Cu}_{3}{\left(BTC\right)}_{2}\right]\right)\times 100\%.$$ (1)

The DOX release profile from the Fe_3_O_4_@[Cu_3_(BTC)_2_]/DOX was investigated *via* dialysis. 3 mg of DOX-loaded nanocomposite was dissolved in 5 ml of a number of buffer solutions with different pHs and placed in a dialysis membrane with MWCO of 14 kDa. The mixture was then dialyzed in 100 ml of PBS at 37°C. At each time point, 2 ml of the release medium was sampled to observe the DOX percentage released in intervals, using UV-Vis with a wavelength of 480 nm. The DOX release percentage was found using the following formula:

$$Er=\frac{Ve\sum_{i=1}^{n-1}Ci+VoCn}{m}$$ (2)

In this formula, Er refers to the DOX release percentage (%); Ve is the volume which was taken out (2 ml); Ci refers to the concentration in μg/mL at time i, wherein i = n − 1; Vo is the total volume of PBS outside the dialysis bag (100 ml); Cn representates the concentration at a certain time (μg/ml); and m refers to the total amount of DOX in Fe_3_O_4_@[Cu_3_(BTC)_2_]/DOX (mg).

Drug release data were plotted and fitted to the drug release kinetics models to propose a release mechanism as follows (Nie et al., 2011):

$$Higuchi\;model:M_{t}\;/M_{\infty }={Kt}^{1/2}$$ (3)

$$Zero-order\;model:M_{t}\;/M_{\infty }=Kt$$ (4)

$$First-order\;model:M_{t}\;/M_{\infty }=1\;–\;e^{-Kt}$$ (5)

The correlation coefficient was then calculated to find the best fit. The release data were also fitted to the Korsmeyer–Peppas model to reveal the release mechanism according to the formula below:

$$Korsmeyer–Peppas\;model:M_{t}\;/M_{\infty }={Kt}^{n}$$ (6)

In all of the above-mentioned formulas, M_t_/M_∞_ shows the drug fraction released at time t, K refers to the rate constant and the exponent “n” shows the drug transport mechanism to evaluate the diffusion mechanism (Reddy et al., 2014) (Mosallanezhad et al., 2022).

**Transverse relaxivity and T**_**2**_ **-weighted images.** The samples with different Fe concentrations (0.00, 0.02, 0.04, 0.06, 0.08, 0.10 mM) were prepared using Fe_3_O_4_@[Cu_3_(BTC)_2_] composites. T_2_-weighted images and transverse relaxivity time (T_2_) of the materials were obtained, using a Siemens Prisma 3.0 T MR scanner (Erlangen, Germany) having a gradient strength of 80 mT/m. The transverse relaxivity (r_2_) of Fe_3_O_4_@[Cu_3_(BTC)_2_] was found by linear fitting of 1/T_2_ *versus* Fe concentration.

***In vitro* MTT assessments.** The MTT assay for evaluation of cytotoxicity was used towards 3 cell lines including 3T3 (mouse embryonic fibroblasts) as normal cells, MCF-7 (breast cancer cell line) and HeLa (human cervical cancer cells) as cancer cell lines. 3T3, MCF-7 and Hela (7×10^3^ cells per well) cells were seeded in 96-well assay plates with 100 µl of culture medium (DEMEM) and placed in an incubator for 24 h. The cells were kept in a 5% CO_2_ at 37 °C and in a humidified incubator. In the next step, 100 μl of various concentrations of Fe_3_O_4_@[Cu_3_(BTC)_2_], Fe_3_O_4_@[Cu_3_(BTC)_2_]/DOX and free DOX were added and placed in the incubator for 24 h. 100 µl of 3- (4, 5dimethylthiazol-2-yl) -2, 5-diphenyl tetrazolium bromide (MTT) (0.5 mg/ml in PBS) were added to the media containing Fe_3_O_4_@[Cu_3_(BTC)_2_], Fe_3_O_4_@[Cu_3_(BTC)_2_]/DOX, and free DOX. The plate was placed in an incubator and cultured for 4 h to achieve the purple formazan product. At the final stage, 50 µl of DMSO was replaced with the medium in each well to dissolve formazan. A BioTek plate reader with a wavelength of 570 nm was used to observe the absorbance of each well. The cell viability percentage was calculated compared with untreated control cells. The results were shown as mean value ±standard deviation (SD).

**Annexin V-FITC** apoptosis **assessment.** The Effect of Fe_3_O_4_@[Cu_3_(BTC)_2_]/DOX and free DOX on apoptosis and necrosis in HeLa cells was studied *via* Annexin V-FITC apoptosis method. Cells were seeded in a 6 well plate (1×10^5^ cells/well) and placed in a CO_2_ incubator at 37°C for 24 h. Cells were treated with Fe_3_O_4_@[Cu_3_(BTC)_2_]/DOX of concentration equivalent to 8 μg/ml of DOX and free DOX in an incubator at a temperature of 37°C for 7 h. Cells were then rinsed twice with PBS and after centrifuging for 5°min, was resuspended in 100 µl Annexin V binding buffer and were placed in an incubator for 20 min with Annexin V-FITC (5 μl) and propidium iodide solution in a dark environment. 400°µl of Annexin V binding buffer was added in FACS tube. Then the cells were observed in a Flow Cytometer. Data analyzed by Flowing Software (Version 2.5.1, Turku Centre for Biotechnology, Finland).

## Results and discussion

**FIGURE 1:**
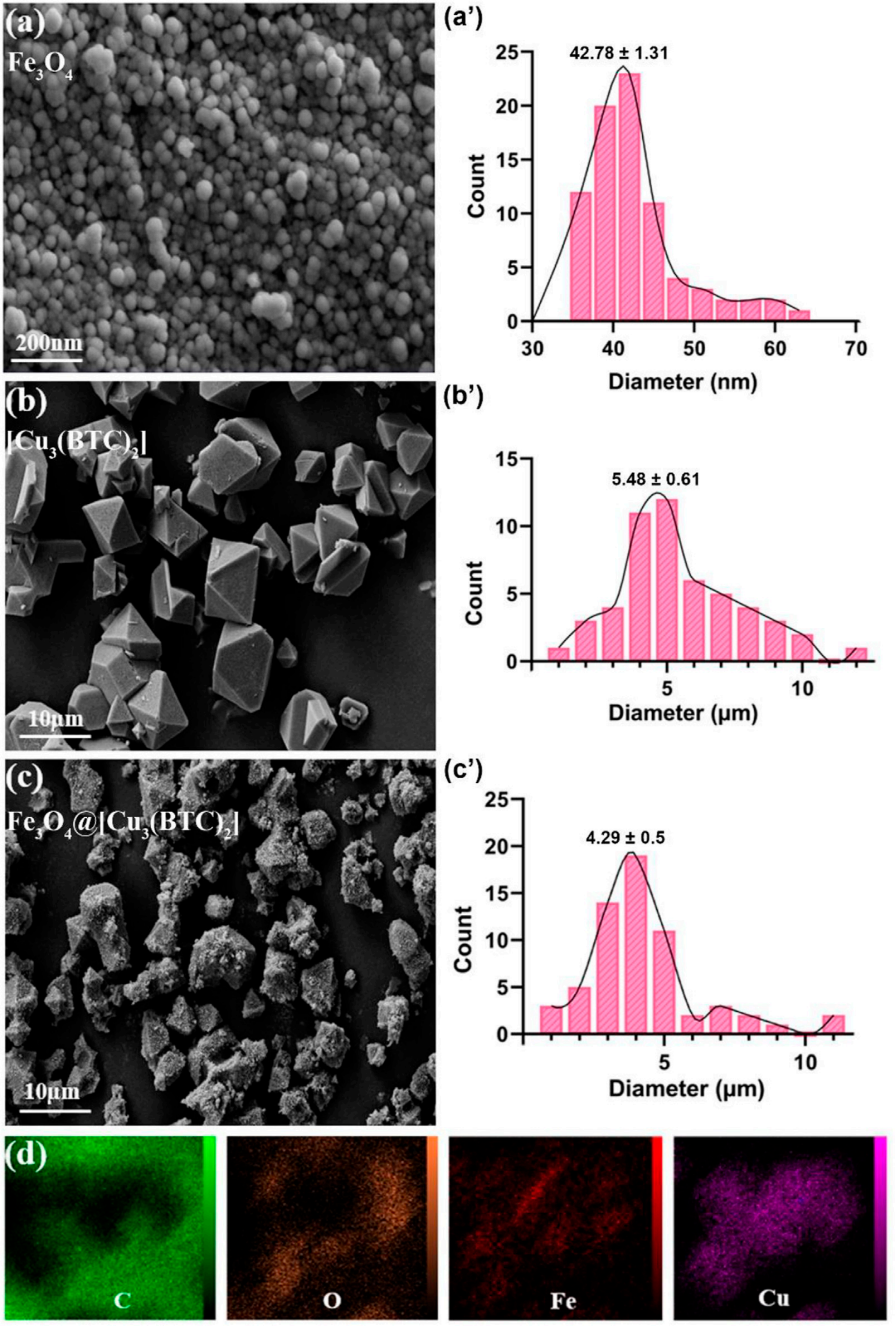
FE-SEM analysis for **(A)** Fe_3_O_4_, **(B)** [Cu_3_(BTC)_2_] and **(C)** Fe_3_O_4_@[Cu_3_(BTC)_2_], respectively. **(D)** The elemental mapping of magnetic Fe_3_O_4_@[Cu_3_(BTC)_2_] nanocomposite.

**FESEM study.** The morphology and structure of the produced samples were studied in this section. According to the FESEM images, Fe_3_O_4_ NPs are mono-dispersed with a spherical shape and an average diameter of 45 nm (Figure 1A). Moreover, size of [Cu_3_(BTC)_2_] was obtained 5.48 µm with an octahedral structure made of dimer Cu paddle wheels linked with BTC (Figure 1B) (Wee et al., 2012). Cu^2+^ ions have weak bindings wherein the residual axial coordination sites are filled by water molecules with a weak bond. The primary building blocks were combined using BTC ligands to produce a 3D octahedral structure with an open-pore system (Schlichte et al., 2004). As seen, Fe_3_O_4_@[Cu_3_(BTC)_2_] also shows an octahedral morphology similar to [Cu_3_(BTC)_2_] together with Fe_3_O_4_ NPs on its surface (Figure 1C). In this regard, Cu^2+^ ions and functional MAA- Fe_3_O_4_ were combined with free state and the [Cu_3_(BTC)_2_] nucleation rate is controlled by manipulating the organic ligand addition speed. The distribution of Fe and Cu elements in the nanocomposite were observed using elemental mapping analysis as well (Figure 1D). The obtained results verified the production of magnetic Fe_3_O_4_@[Cu_3_(BTC)_2_] nano/microcomposite.

**FIGURE 2:**
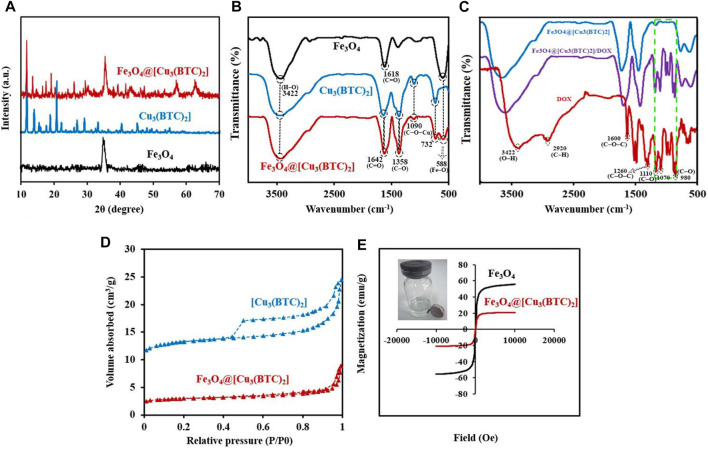
**(A)** PXRD patterns for Fe_3_O_4_, [Cu_3_(BTC)_2_] and Fe_3_O_4_@[Cu_3_(BTC)_2_]. **(B)** FT-IR analysis for the materials produced: Fe_3_O_4_, [Cu_3_(BTC)_2_] and Fe_3_O_4_@[Cu_3_(BTC)_2_]. **(C)** FT-IR spectra for Fe_3_O_4_@[Cu_3_(BTC)_2_], Fe_3_O_4_@[Cu_3_(BTC)_2_]/DOX and free DOX **(D)** N_2_ adsorption*-*desorption isotherms of [Cu_3_(BTC)_2_] and Fe_3_O_4_@[Cu_3_(BTC)_2_]. **(E)** VSM analysis for Fe_3_O_4_ and Fe_3_O_4_@[Cu_3_(BTC)_2_].

**X-ray diffraction study.** The crystal structure of the obtained composite was studied *via* powder X-ray diffraction (PXRD) (Figure 2A). The Fe_3_O_4_ diffraction peaks were appeared at 2θ = 34.70° (311), 41.45° (400), 51.75° (422), 56.94° (511) and 62.45° related to the (440) plan (JCPDS No.19–0629, magnetite) (Yang et al., 2017). The resulting XRD peaks of Fe_3_O_4_@[Cu_3_(BTC)_2_] belong to the crystalline Fe_3_O_4_ and [Cu_3_(BTC)_2_] (Zeng et al., 2014), suggesting crystalline structure formation of the Fe_3_O_4_@[Cu_3_(BTC)_2_] composite. Moreover, no impurities were detected according to the peaks. Or there is not a physical mixture of two separate phases of Fe_3_O_4_ and [Cu_3_(BTC)_2_].

**FT-IR spectra.** The FT-IR spectra of Fe_3_O_4_ nanospheres, [Cu_3_(BTC)_2_] and the magnetic nanocomposite Fe_3_O_4_@[Cu_3_(BTC)_2_] are illustrated in Figure 2B. The FTIR spectrum of [Cu_3_(BTC)_2_] shows the characteristic peaks at 732 and 1090 cm^−1^ related to the C−O−Cu stretching vibrations, and two sharp peaks at 1358 and 1642 cm^−1^ attributed to the C–O and C=O stretching vibrations, respectively. Moreover, a broad band is observed about 3500 cm^−1^ which could be assigned to O−H stretching vibration or adsorbed water. MAA-Fe_3_O_4_ NPs exhibits the characteristic FT-IR peak specially at 588 belonged to the Fe–O vibration, a small peak at 2693 cm^−1^ related to S−H stretching vibration and a sharp peak at 1618 cm^−1^ as well as a broad peak at 3422 cm^−1^ attributed to the starching C=O and H–O vibrations of MAA located on the surface of Fe_3_O_4_, respectively (Bandosz and Petit, 2011; Zeng et al., 2013). Notably, the peaks at 1090 and 732 cm−1 (C−O−Cu), as well as the other 588 (Fe–O), 1358 (C–O) and 1642 cm^−1^ (C=O) intensified in the FT-IR spectrum of Fe_3_O_4_@[Cu_3_(BTC)_2_] indicate the successful formation of Fe_3_O_4_@[Cu_3_(BTC)_2_] composites (Varghese et al., 2022). The peak intensity was lower in Fe_3_O_4_@[Cu_3_(BTC)_2_] due to the immobilization caused by coating [Cu_3_(BTC)_2_].

The IR spectra of Fe_3_O_4_@[Cu_3_(BTC)_2_] as a new proposed pH-responsive platform was also compared before and after adding DOX as a commercially anticancer drug model. The FT-IR spectrum of DOX illustrated characteristics peaks including C–O stretching primary alcohol (980 cm^−1^), C–O stretching secondary alcohol (1070 cm^−1^), C–O stretching tertiary alcohol (1110 cm^−1^), C–O–C stretch (1260 cm^−1^), C=O stretch (1600 cm^−1^), C=O stretch ketone (1730 cm^−1^), C–H stretch aromatic (2920 cm^−1^), and O–H and N–H stretches (2500–3600 cm^−1^). As shown in Figure 2C, the characteristic sorption bonds at 980, 1070, and 1110 cm^−1^ of DOX labelled with green dash rectangle in the spectra of Fe_3_O_4_@[Cu_3_(BTC)_2_]/DOX confirmed the successful loading of DOX in the Fe_3_O_4_@[Cu_3_(BTC)_2_] nanocomposite (Figure 2C).

**Nitrogen adsorption–desorption isotherms.** In order to calculate the surface area and study the porosity of the materials and pore volumes, nitrogen adsorption‐desorption isotherms were recorded (Figure 2D). As seen, the adsorption‐desorption isotherms intermediate between type I and IV were observed for Fe_3_O_4_@[Cu_3_(BTC)_2_] as well as [Cu_3_(BTC)_2_]. This indicates that there is the copresence of micropores and mesopores in materials (Ke et al., 2014) (Zhang et al., 2016). Table 1 shows the BET pore volumes and surface areas. The BET total pore volume and surface area of Fe_3_O_4_@[Cu_3_(BTC)_2_] were 0.236 × 10^–6^ m^3^g^−1^ and 301.69 m^2^g^−1^, respectively, which is lower than that of [Cu_3_(BTC)_2_] MOF (Table 1). This can be due to the presence of Fe_3_O_4_ NPs on the octahedral structure of MOF. Average pore size was 3.2 nm which was calculated from N_2_ isotherm desorption using BJH method. Really, the high porosity and specific surface area may result in multiple channels for drug loading.

**TABLE 1:** BET surface area, total pore volume, and pore diameter of [Cu_3_(BTC)_2_] and Fe_3_O_4_@[Cu_3_(BTC)_2_] based on N_2_ adsorption isotherms at 77K.

Samples	BET surface area (m2g−1)	Total pore volume (x106m3g−1)	Pore diameter (nm)
[CU3(BTC)2]	800	0.4	4.2
Fe3O4at[Cu3(BTC)2]	301.69	0.2	3.2

**VSM measurements**. The magnetic hysteresis loops of magnetic nanocomposite Fe_3_O_4_@[Cu_3_(BTC)_2_] and Fe_3_O_4_ nanospheres were measured at 300 K by a VSM with a field ranging from -20000 to +20,000 Oe; wherein the results are depicted in Figure 2E.

The Ms (saturation magnetization) values for Fe_3_O_4_@[Cu_3_(BTC)_2_] nanoparticles were 20.87 emu/g which were lower than that of Fe_3_O_4_ nanospheres (55.58 emu/g) (Figure 2E). A large surface area to volume ratio magnetic anisotropy energy of nanocomposite caused that the magnetization reduction of nanocomposite could be comparable to the thermal energy. The thermal fluctuations can significantly reduce the magnetic moment in a specific field (Spaldin, 2010; Ghosh et al., 2011). The high Ms value allows the Fe_3_O_4_@[Cu_3_(BTC)_2_] nanocomposites to be used in MRI, magnetic targeted drug carriers, adsorption and many other applications.

**FIGURE 3:**
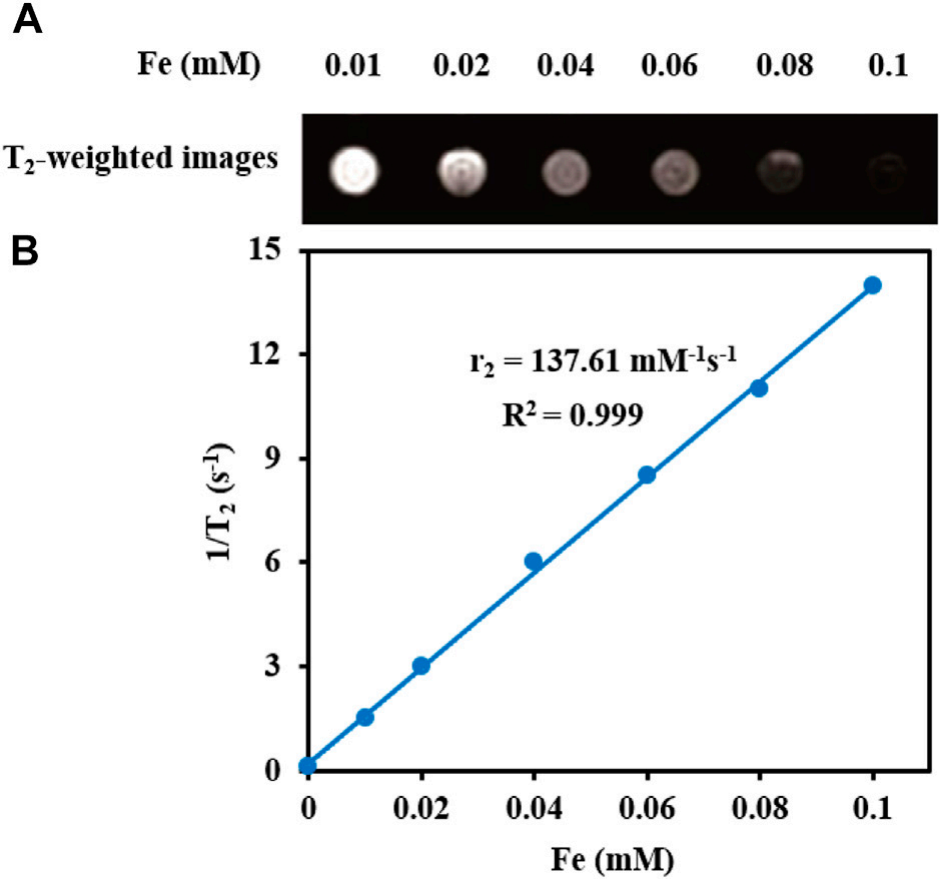
**(A)** T_2_-Weighted MRI images of Fe_3_O_4_@[Cu_3_(BTC)_2_] at various Fe concentrations **(B)** Plot of inverse transverse relaxation time (1/T_2_) *versus* Fe concentrations. The slope indicates the relaxivity value, r_2_.

**T**_**2**_ **-weighted MRI.** The application of Fe_3_O_4_@[Cu_3_(BTC)_2_] nanocomposite as an MRI contrast agent is evaluated using T_2_-weighted MR imaging with a 3T clinical MRI instrument. To evaluate the T_2_ effect, the concentration-dependent darkening of the Fe_3_O_4_@[Cu_3_(BTC)_2_] nanocomposite was recorded and shown in Figure 3. Accordingly, the T_2_ contrast depended on the concentration of Fe. Figure 3 shows T_2_ relaxation rates (1/T_2_) of Fe_3_O_4_a@[Cu_3_(BTC)_2_] nanocomposites with different concentrations of iron. Moreover, the transverse relaxivity (r_2_) of Fe_3_O_4_@[Cu_3_(BTC)_2_] was reported 137.61 mM/s. Thus, the results indicate that the Fe_3_O_4_@[Cu_3_(BTC)_2_] composites with very low concentrations may be applicable as MRI contrast agents. Furthermore, the r_2_ value of the prepared Fe_3_O_4_@[Cu_3_(BTC)_2_] was much higher than that of PDA-ICG-PEG/DOX (Mn) (39.2 mM/s) (Dong et al., 2016) and clinical Fe-based T_2_-weighted contrast agents such as ferumoxide (98.3 mM/s) and ferumoxsil (72 mM/s). According to the results, Fe_3_O_4_@[Cu_3_(BTC)_2_] could be applicable as a strong MRI contrast agent in T_2_-MR imaging.

***In vitro* drug load/release of nanomagnetic Fe**_**3**_ **O**_**4**_ **at[Cu**_**3**_ **(BTC)**_**2**_ **].** Because of the unique properties of the as-prepared magnetic MOF composite, we designed a targeted drug delivery nano/microcarrier for cancer treatment based on applying and loading DOX into magnetic composite. In this sense, DOX was added to Fe_3_O_4_@[Cu_3_(BTC)_2_] in PBS for 24 h at a pH of 7.4. Then, the unloaded DOX was removed to obtain Fe_3_O_4_@[Cu_3_(BTC)_2_]/DOX and as-above mentioned the IR spectra were used to confirm DOX loading in Fe_3_O_4_@[Cu_3_(BTC)_2_] composite (Figure 2C). With the appearance of peaks related to DOX in as-prepared MOF composite, the drug loading of Fe_3_O_4_@[Cu_3_(BTC)_2_]/DOX was calculated and found around 40.5 wt%. This high drug loading of Fe_3_O_4_@[Cu_3_(BTC)_2_] nanocomposite is due to the high surface area of [Cu_3_(BTC)_2_] and the interactions between DOX and [Cu_3_(BTC)_2_]. Interactions include π-π stacking effect between the [Cu_3_(BTC)_2_] aromatic porous walls and the DOX aromatic anthracycline, hydrogen bonding between the [Cu_3_(BTC)_2_] carboxyl groups and the DOX oxygen atoms, electrostatic interactions, Van Der Waals forces, and coordination bonds (Horcajada et al., 2012). The coordination bonds between DOX deprotonated hydroxyls and the Cu sites in [Cu_3_(BTC)_2_] had a major impact in drug delivery (Zhao et al., 2016).

**FIGURE 4:**
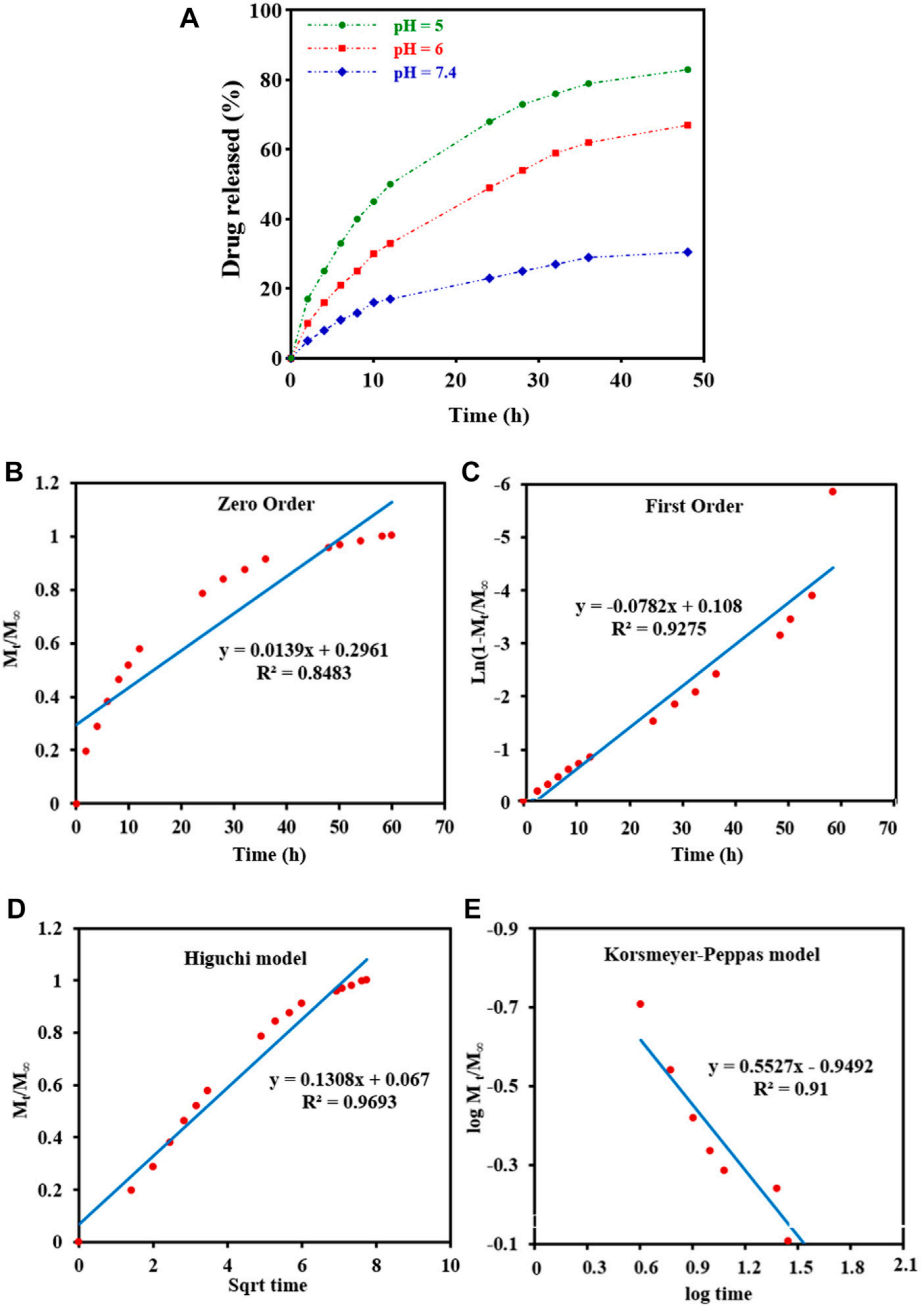
**(A)** *In vitro* DOX release of the Fe_3_O_4_@[Cu_3_(BTC)_2_]/DOX at different pH 5,6, and 7.4. **(B–E)** Drug release data fitted to various kinetic models. **(B)** Zero order; **(C)** First order; **(D)** Higuchi model; and **(E)** Korsmeyer-Peppas drug diffusion model.

DOX-release experiments were performed in PBS buffer solutions (pH 5,6 and 7.4 at 37°C) and characterized by UV-Vis to evaluate the drug delivery properties of DOX-loaded Fe_3_O_4_@[Cu_3_(BTC)_2_] in different physiological environments (Figure 4A). As can be seen in Figure 4A, the released amount of drug after 48 h was about 83% at a pH of 5.0, and around 30.5% at a pH of 7.4. Therefore, drug release from Fe_3_O_4_@[Cu_3_(BTC)_2_]/DOX was slower at a pH of 7.4 in comparison with a pH of 5, due to the electrostatic interaction between framework rings and drug molecules with pH change. According to the results, small amounts of DOX are released at normal cell pH and therefore the normal cells are not affected by the undesirable side effects of the drug; while, in an acidic pH (cancer cells environment), the drug release is increased to eliminate tumor cells. Therefore, Fe_3_O_4_@[Cu_3_(BTC)_2_] nano/microcomposite had pH-responsive properties which may be used to adjust DOX release and prevent premature release in physiological conditions. Notably, two variation of drug molecules existed in the release process. The drug was released with a high rate during the first 10 h followed by gradual drug dissolution. The guest molecules near the boundaries had host–guest interactions, such as π–π interactions and hydrogen bonds between the framework organic part and the DOX aromatic rings. Meanwhile, drug molecules far from the walls had guest-guest interactions. The molecules with weak bonds were dissolved in the primary stage of the delivery (Lin et al., 2016).

To study drug release from nanoparticles *in vitro*, the results are verified with a mathematical model (Gandhi et al., 2014) (Figure 4 b-e) (Table 2). As shown in Figure 4D, Higuchi model exhibited the best fit of all data points (up to 100% of release curves), since R_h_ was the best match compared to other coefficients. The Higuchi release model is represented as:

$$M_{t}/M_{\infty }=K_{h}t^{1/2}$$

Wherein M_t_ refers to the DOX release amount at time t, M_∞_ shows the maximum DOX release, and k_h_ is the Higuchi constant for DOX release. *R* ^2^ was reported to be 0.9693, according to an “n” value (diffusion exponent) in the range of 0.45–0.89, found from the Korsmeyer–Peppas model. Therefore, it can be concluded that the DOX release was non-Fickian model, with an anomalous transport diffusional release, suggesting both swelling- and diffusion-controlled drug release.

**TABLE 2:** Kinetic models for drug release.

Kinetic models	Fitting equation	R2	K (h−1)
Mt/M∞ = Kt	Mt/M∞ = 0.0139t	0.848	0.0139
Mt/M∞ = 1—e−Kt	Mt/M∞ = 1—e−0.0782t	0.927	0.0782
Mt/M∞ = Kt1/2	Mt/M∞ = 0.13t1/2	0.969	0.130
Mt/M∞ = Ktn	Mt/M∞ = 0.11t0.552	0.910	0.11

**FIGURE 5:**
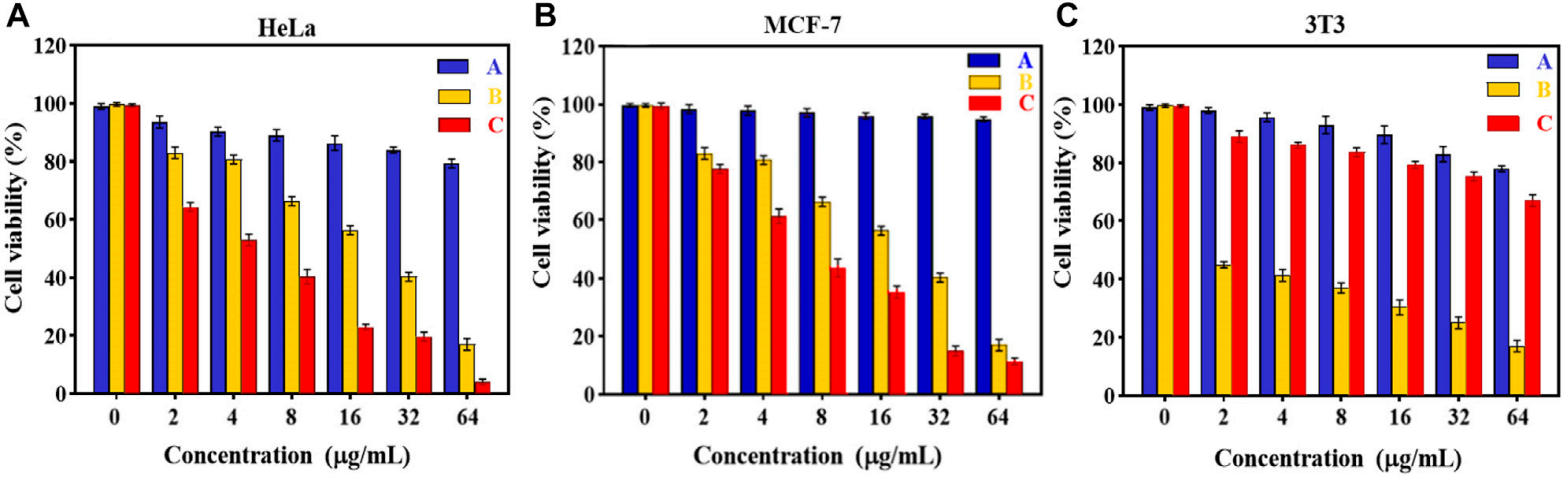
Cell viability of **(A)** HeLa, **(B)** MCF-7 and **(C)** 3T3 cells after incubating with various amounts of **(A)** Fe_3_O_4_@[Cu_3_(BTC)_2_] and **(B)** free DOX, **(C)**Fe_3_O_4_@[Cu_3_(BTC)_2_]/DOX for 24 h (Data were given as mean ± SD, n = 3).

***In vitro* cytotoxicity tests.** The effect of Fe_3_O_4_@[Cu_3_(BTC)_2_] and Fe_3_O_4_@[Cu_3_(BTC)_2_]/DOX on the viability of two cancer cell lines, human cervical cancer HeLa and breast cancer MCF-7 cells (Madej et al., 2022), was investigated in the range of 2.0–64 μg/ml. The results showed significant cell survival due to the low cytotoxicity of Fe_3_O_4_at[Cu_3_(BTC)_2_], so that the viability remained above ca. 80% for HeLa cells and 95% for MCF-7 cells after 24 h of incubation time. This result verified the high biocompatibility of Fe_3_O_4_@[Cu_3_(BTC)_2_] even with a high concentration 64 μg/ml of magnetic nano/microcomposite (Figure 5). The inhibitory effect of DOX released from Fe_3_O_4_at[Cu_3_(BTC)_2_]/DOX on the growth of HeLa and MCF-7 cells was also examined and compared to the cytotoxicity of free DOX as a control experiment. The results depict that Fe_3_O_4_@[Cu_3_(BTC)_2_]/DOX was much higher cytotoxic to cancer cell lines than free DOX (Figures 5C,D). The IC_50_ of Fe_3_O_4_@[Cu_3_(BTC)_2_]/DOX on HeLa cells (4.45 μg/ml) and MCF-7 cells (6.8 μg/ml) was obtained about 4.0 and 2.6 times more than that of free DOX state (17.74 μg/ml), respectively (Table 3). This observation can be attributed to more uptake of Fe3O4at[Cu3(BTC)2]/DOX by cancer cells, which allows pH-responsivity mediated specific endocytosis and thus a growth inhibition and cell death. Consequently, the DOX released from DOX-loaded Fe3O4@[Cu3(BTC)2] possess an anticancer potential and tumor-targeting activity (Shamsipur et al., 2018).

**TABLE 3:** IC50 values on HeLa, MCF-7 and 3T3 cells were calculated using non-linear regression analysis in Graph Pad Prism 8.0.2 software (+SD; n = 3).

Cell lines	IC_50_(μg/mL)		
	Fe_3_O_4_@[Cu_3_(BTC)_2_]	Fe_3_O_4_@[Cu_3_(BTC)_2_]/DOX	DOX
HeLa	3109	4.45	17.74
MCF-7	7530	6.8	18.01
3T3	410.5	431.5	1.45

To further confirm the pH responsivity of proposed magnetic nano/microcomposite, one normal cell line, mouse embryonic fibroblasts 3T3 cells was selected and incubated with various concentrations of the as-synthesized magnetic composite same as above to investigate their cell viability. As shown in Figures 5A, B, both Fe3O4at[Cu3(BTC)2] and Fe3O4at[Cu3(BTC)2]/DOX displayed low cytotoxic activity at even high concentrations, since the viability remained above ca. 80% by composite and 70% by Dox-loaded composite after 24 h incubation time, revealing that both nano/microcomposites are of high biocompatibility toward normal cells and thus promising ability for use in tumor treatment with a minimum side effect. Fe_3_O_4_@[Cu_3_(BTC)_2_]/DOX was also showed a much lower cytotoxicity to normal 3T3 cells compared to free DOX (∼3.5 times). This further demonstrates the pH responsivity of developed nano/micro magnetic composite because the structural properties of [Cu3(BTC)2] MOFs play an absolutely crucial role in their toxicity to cells. Overall, it can be said that the proposed nano/microcomposte magnetic [Cu3(BTC)2] MOF can act as a selective dual functional targeted nanocarrier-assisted pH-responsive drug delivery system with potential of **MRI** property. Therefore, the Fe_3_O_4_@[Cu_3_(BTC)_2_]/DOX DDS may be a potential substitute for targeted cancer therapy, as the system uses lower dose of DOX to achieve similar cytotoxicity to tumor cells, while decreases the side effects on normal cells (Shahrousvand et al., 2022).

## Annexin V-FITC apoptosis assay

**FIGURE 6:**
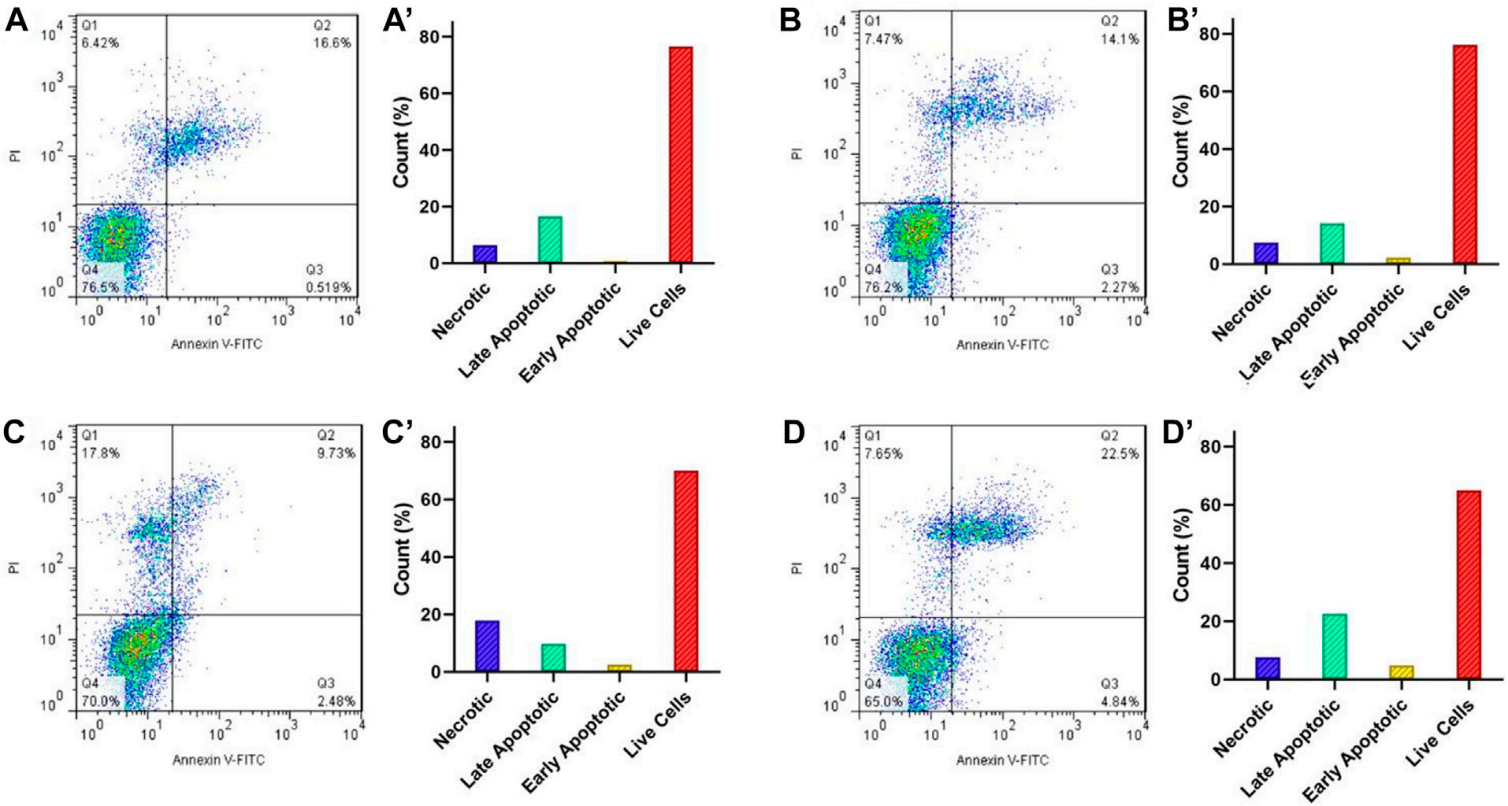
Flow cytometry analysis of the apoptotic and necrotic HeLa cells at pH 7.4 after 6 h incubation with the control group **(A)**, Fe_3_O_4_@[Cu_3_(BTC)_2_] **(B)**, free DOX **(C)**, and Fe_3_O_4_@[Cu_3_(BTC)_2_]/DOX **(D)**. The results are expressed as mean ± standard deviation (n = 3). Q1−Q4 represent the necrotic, late apoptotic, early apoptotic, and live cells, respectively.

Induction of apoptosis is proposed as the main the mechanism in most anticancer drugs (Chen et al., 2010). For this reason, the apoptotic impact of Fe_3_O_4_@[Cu_3_(BTC)_2_]/DOX on the HeLa cell line was evaluated *via* flow cytometry using PI and Annexin-V FITC double-staining method (Figure 6). For this purpose, HeLa cells were treated with 8 μg/ml Fe_3_O_4_@[Cu_3_(BTC)_2_]/DOX, free DOX and Fe_3_O_4_@[Cu_3_(BTC)_2_] for 12 h and after that the percentages of necrotic, early and late apoptotic, and live cells after treatments were investigated. According to the results, cells exposed to free DOX (12.21%) had a lower apoptosis percentage compared to when HeLa cells exposed to Fe_3_O_4_at[Cu_3_(BTC)_2_]/DOX (27.34%). As a conclusion, it can be mentioned that Fe_3_O_4_at[Cu_3_(BTC)_2_]/DOX triggered a significantly higher amount of apoptosis in the HeLa cells compared to free DOX state.

## Conclusion

In summary, a multifunction magnetic nano/microcomposite has been synthesized using a novel convenient method, consisting [Cu_3_(BTC)_2_] as a targeted drug delivery system with enhanced antitumor efficacy and Fe_3_O_4_ as T_2_-weighted MR imaging agent. DOX was loaded into the prepared magnetic nanocomposite as a model drug, which provided high drug loading capacity. Moreover, according to *in vitro* studies, the DOX released from Fe_3_O_4_@[Cu_3_(BTC)_2_]/DOX was 83.0% at a pH of 5 while this amount reduced to 30.5% at a physiological pH (7.4). These results showed that multifunctional magnetic nano/microcomposite had a high DOX release rate under acidic conditions of tumor cells, and a low release rate at a physiological condition of healthy cells with a pH 7.4. Additionally, the cytotoxicity assay studies demonstrated that the magnetic composites have lower toxicity and higher biocompatibility, while DOX-loaded Fe_3_O_4_@[Cu_3_(BTC)_2_] caused higher toxicity in cancer cells and lower toxicity in normal cells compared to free DOX due to the pH-responsive behavior. Furthermore, the transverse relaxivity of Fe_3_O_4_@[Cu_3_(BTC)_2_] was found to be 137.61 mM/s, showing its high ability to be used as a contrast agent for MR imaging. Further, flow cytometry was used to study the apoptosis amount induced by the Fe_3_O_4_@[Cu_3_(BTC)_2_]/DOX. Really, the Fe_3_O_4_@[Cu_3_(BTC)_2_] magnetic nano/microcomposites exhibited a significantly higher drug loading capacity, high transverse relaxivity, and lower cytotoxicity. Therefore, it can be said that developed Fe_3_O_4_@[Cu_3_(BTC)_2_] magnetic nano/microcomposites may be used as a strong potential drug delivery system in biomedicine and as a promising theranostic agent for MR imaging.

## Data Availability

The raw data supporting the conclusions of this article will be made available by the authors, without undue reservation.
